# Perception of cure and quality of life in anal cancer survivors

**DOI:** 10.2340/1651-226X.2025.44133

**Published:** 2025-10-15

**Authors:** Nina Fanni, Eva Haglind, Sofia Heyman, Mia Johansson, Ying Li, Anna Perman, Eva Angenete

**Affiliations:** a Department of Surgery, SSORG-Scandinavian Surgical Outcomes Research Group, Institute of Clinical Sciences, Sahlgrenska Academy, University of Gothenburg, Gothenburg, Sweden; b Department of Anaesthesiology, Intensive Care Medicine and Pain Medicine, Sahlgrenska University Hospital, Gothenburg, Sweden; c Department of Surgery, Sahlgrenska University Hospital, Gothenburg, Sweden; d Department of Oncology, Institute of Clinical Sciences, Sahlgrenska Academy, University of Gothenburg, Gothenburg, Sweden; e Department of Oncology, Sahlgrenska University Hospital, Gothenburg, Sweden; f School of Public Health and Community Medicine, Institute of Medicine, Sahlgrenska Academy, University of Gothenburg, Gothenburg, Sweden

**Keywords:** anus neoplasms, prognosis, treatment expectations, patient reported outcome measures, fear of recurrence

## Abstract

**Background and purpose:**

Anal cancer is treated with chemoradiotherapy and with salvage surgery in case of local failure. Curation rate depends on tumour stage but is overall high (80–90%).

This study explored anal cancer survivors’ perception of being cured and possible association with Quality of Life (QoL). Secondary aims were to explore fear of recurrence and if perception of cure changed over time or differed depending on tumour stage.

**Patients/material and methods:**

The ANal CAncer study comprises a cohort of patients diagnosed with anal cancer in Sweden between 2011 and 2013 (*n* = 388). Participants filled out a study-specific questionnaire 3 and/or 6 years after diagnosis (*n* = 205). In this sub-study, only patients treated with curative intent were included. The association between perception of cure and QoL was analysed using logistic regression. Descriptive analyses were performed regarding secondary aims.

**Results:**

A high proportion (80%) of the patients were either moderately or very sure of cure across all tumour stages, in both early and advanced tumour stages, and with no obvious improvement over time. Despite this 42% experienced fear of recurrence 6 years after diagnosis. A strong perception of cure was correlated with high QoL.

**Interpretation:**

Even though most patients had a perception of being cured, several patients irrespective of tumour stage, felt unsure of cure and feared recurrence years after successful treatment. A strong perception of cure was also shown to be associated with a high QoL. We suggest that improved counselling could enhance the patient’s perception of cure and to possibly improve QoL.

## Introduction

Anal cancer is treated with chemoradiotherapy. Most patients with locoregional disease are cured and in case of residual disease or local recurrence, salvage surgery may be required [1]. A recent national Swedish cohort study reported a 3-year overall survival of 85% and in a previous randomised trial from the UK the 3-year anal cancer specific survival has been estimated to be 88% [2, 3]. Recurrences are few and up to 82% of them occur during the first 3 years after treatment [4]. From the physician’s perspective, the probability of cure after anal cancer therapy is high. However, little is known about the patient’s perception of being cured.

Prognostic awareness refers to a patient’s understanding of the severity of the illness and the likelihood of cure and survival [5]. This has been explored in patients with incurable cancer. Interestingly, 30–80% of patients treated in a palliative setting believed they were receiving chemotherapy with a curative intent [6–8]. Furthermore, a correctly perceived poor prognosis has been correlated with a decreased Quality of Life (QoL), although results are conflicting [7–11].

In patients receiving curative treatment for different cancer forms, an accurate expectation of cure is reported to be 80–90% [12, 13]. To our knowledge, it has not been studied how the cancer patient’s understanding of treatment intent and the probability of cure affects QoL in the curative setting.

Since patients treated curatively for anal cancer carry a very good prognosis with almost negligible risk of recurrence after 3 years, they make a particularly suitable group for exploration of perception of cure and fear of recurrence.

QoL in anal cancer survivors has been described in relation to treatment-related toxicities. Chemoradiotherapy is associated with a negative effect on bowel, urinary and sexual function. This in turn seems to deteriorate QoL [14, 15]. More specifically, patient-reported ‘bother’ of these bodily dysfunctions has been associated with poorer QoL [16].

However, QoL is a multi-dimensional concept that might also be affected by an incorrect perception of cure, and if so, it is possible that we could enhance it with improved information and counselling.

Fear of recurrence seems to be common among cancer survivors for many years after diagnosis and to be negatively associated with global QoL but also affecting some subdomains [17–19]. This could possibly be related to the perception of cure, but it might also exist in patients who initially were certain to be cured.

The aim of this study was to investigate the impact of the patient’s perception of cure on patient-reported QoL 3 and 6 years after receiving anal cancer treatment with curative intent. Our hypothesis was that the patient’s certainty of cure was associated with a high QoL. Another hypothesis was that perception of cure depends on tumour stage and time elapsed since treatment. Finally, we wanted to explore fear of recurrence 6 years after treatment.

## Patients/material and methods

### Study design

ANal CAncer study (ANCA) is a Swedish national cohort study of patients with anal cancer, who were diagnosed between 1 January 2011 and 31 December 2013. The aims of this longitudinal study were to explore QoL and functional outcome 3 and 6 years after diagnosis. For this specific sub-study, we analysed selected data from all patients who had responded to a questionnaire at 3 and/or 6 years (*n* = 205). None of the responders were treated with a palliative intent.

### Data collection

All patients with a diagnosis of squamous cell carcinoma of the anus were identified from the Swedish Cancer Register at the Swedish National Board of Health and Welfare.

All patients alive were contacted to obtain informed consent for study participation. After given consent, clinical data were gathered from medical records and from the National Patient Register at the National Board of Health and Welfare. Data collection followed a standardised procedure using a pre-specified clinical record form (CRF). Determination of tumour staging (according to American Joint Committee on Cancer/Union for International Cancer Control, seventh edition) and treatment strategy was performed using medical records. This cohort has been described in detail in a previous publication [20].

### Questionnaire

A study-specific questionnaire was developed (since no validated disease-specific existed at that time) based on information retrieved from in-depth interviews with patients as well as information from an expert panel. The content was tested for face-validity on patients. The method used for the development of the questionnaire has been described in detail elsewhere [21, 22].

The questionnaire consisted of 260 questions aiming to mainly investigate effects of treatment on a group of anal cancer survivors. The 29-item Sense of Coherence scale (SOC-29) was also integrated in the questionnaire [23].

Participants were invited to complete this questionnaire 3 and 6 years after diagnosis. After being reached by telephone the participants received the questionnaire through mail. Two weeks later, a post card with a thank you note and if necessary, a reminder was sent out. If this didn’t yield a response within 2 weeks, a last telephone call was made to remind the patient about the questionnaire.

For the purpose of this sub-study, questions relevant for the outcome measures and the possible explanatory variables were selected. Other outcomes have also been published before [16, 24, 25].

### Outcome measures and possible explanatory variables

The primary endpoint QoL was measured through the question *How would you describe your QoL in the past month?* at 3 and 6 years using a Likert scale from 0 to 6 with 0 = *no QoL* and 6 = *the best possible QoL*. According to previous studies the responses were dichotomised to *low QoL* (0–4) or *high QoL* (5–6) [16, 21].

Patients reported perception of cure at 3 and 6 years answering the question *How sure are you today that your anal cancer has been cured?* The four response categories were *not at all sure*, *somewhat sure*, *moderately sure* and *very sure*.

Potential explanatory variables for QoL are mentioned below and were chosen based on clinical experience and previous publications.

Bother due to bowel function was evaluated through the question How would you feel if this last month’s bowel impairment was to remain the same for the rest of your life? Five response categories were available: (1) Not relevant, I haven’t had any bowel impairment the last month, (2) It wouldn’t bother me at all, (3) It wouldn’t bother me slightly, (4) It would bother me moderately, and (5) It would bother me very much. As in a previous publication, we chose to classify the responses into following three categories: no bother (1), minor bother (2 + 3) and major bother (4 + 5) [16].

SOC was measured by the scale SOC-29 [23]. Each item in the instrument is evaluated by a seven-point Likert scale. The total score reflects the level of SOC with a higher value indicating a better capacity to react to stressful situations in life [23]. Results from earlier studies have reported an association between a high SOC and a better QoL, including patients with breast and rectal cancer [26–28].

Depression was evaluated using the single-item question *Would you call yourself depressed?* Possible responses were (1) *no*, (2) *yes* or (3) *I don’t know*. In accordance with a previous study that has validated this question in relation to Hospital Anxiety Depression Scale, the response categories were dichotomised to either *no* (1) or *yes* (2 + 3) [29].

Fear of recurrence was only measured in the 6-year questionnaire with *Are you afraid that the anal cancer will return?* Possible response options were *not applicable (my cancer is not cured)*, *no*, *yes* and *I don’t know*.

Recurrence was defined as *yes*, *no* or *unknown* using information from medical records and the questionnaires. Patient reported recurrence was identified from the question *If you had a recurrence of your cancer, where in your body was it found?* The patients could choose from the categories: (1) *not applicable, I don’t have a recurrence*, (2) *not applicable, I don’t know if I have a recurrence*, (3) *where the anal canal used to be or in the pelvis*, (4) *in the liver*, (5) *in the lungs*, (6) *in the skeleton* and (7) *other*.

### Statistical analysis

All analysis was performed according to a prespecified analysis plan.

Descriptive analysis of data from this cohort was performed. Data are presented as frequencies (percentages described as round numbers) or median values with interquartile range (IQR).

The primary outcome was QoL, dichotomised as *low* or *high*. The key exposure variable was perception of being cured, modelled as a categorical variable with four levels: *not at all sure*, *somewhat sure*, *moderately sure*, and *very sure*. We used a mixed-effects logistic regression to analyse combined data from 3- and 6-year follow-up.

The model was further adjusted for: bother due to bowel function (*no bother*, *minor bother*, *major bother*), SOC, depression (*yes/no*) and recurrence (*yes/no*). We reported both unadjusted and adjusted odds ratios (ORs) with their corresponding 95% confidence intervals (CIs). We initially attempted to estimate risk ratios rather than ORs, as the outcome prevalence was not rare, using a mixed-effects log-binomial model. However, the model failed to converge.

## Results

**Figure 1:**
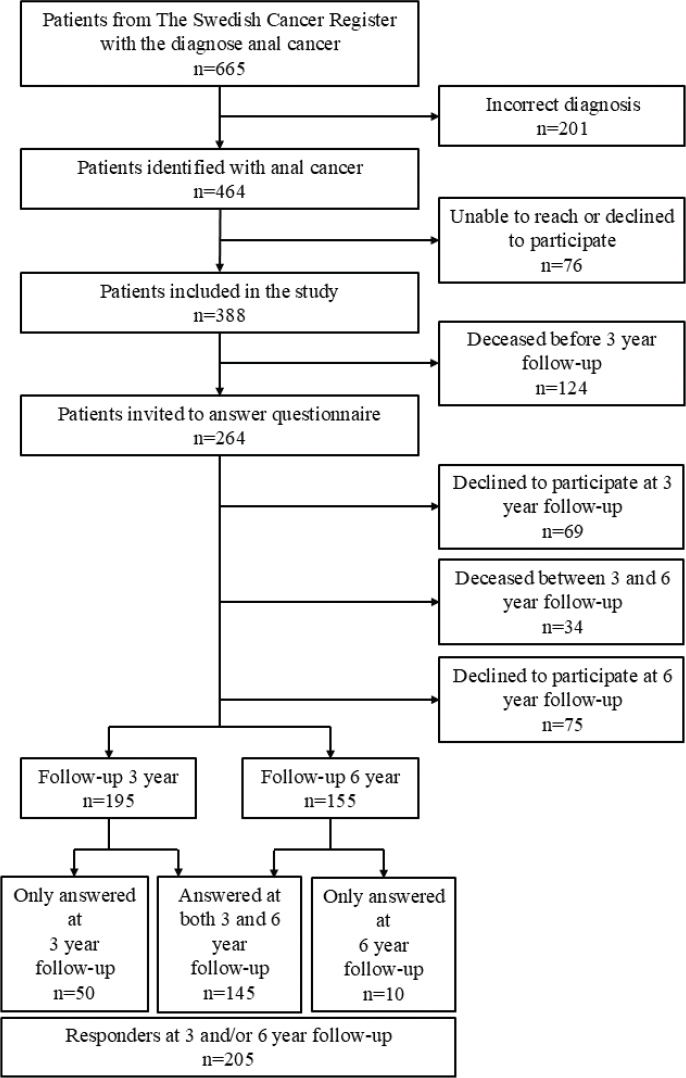
Flowchart ANal CAncer-study.

The study included 388 patients. At 3 years from diagnosis, 264 individuals were alive and invited to respond to a questionnaire, of which 195 patients (74%) replied. By the time of 6-year follow-up, 155 out of 230 patients alive (67%) answered the questionnaire (Figure 1). Ten individuals declined participation at the 3-year follow-up but returned the 6-year questionnaire. In all, results from 205 survivors who responded to at least one of the questionnaires were analysed. The treatment strategy was clearly defined as curative for 203 out of 205 responders (for details regarding treatment modalities see Supplementary Table).

Median age among responders was 64 years (Table 1). Most were female (78%), and in a relationship (65% at 3 years vs. 49% at 6 years). The most frequent comorbidity was cardiovascular disease (13%). Recurrence was identified in 28/205 (14%) of the responders, with 13 cases of local recurrence only. Non-responders compared to responders were older, more often men and had more advanced tumour stage and comorbidity (see Supplementary Table).

**Table 1:** Patient and tumour characteristics.

Characteristic	Cohort in total *n* = 388	Responders 3 and/or 6 years *n* = 205
**Age, median (IQR)**	66 (58, 75)	64 (58, 71)
**Sex, *n* (%)**		
Male	107 (28)	46 (22)
Female	281 (72)	159 (78)
Marital status 3 years*, *n* (%)		
In a relationship		133 (65)
Not in a relationship		62 (30)
Missing		10 (5)
Marital status 6 years*, *n* (%)		
In a relationship		101 (49)
Not in a relationship		53 (26)
Missing		51 (25)
Educational level*, *n* (%)		
Primary and lower secondary		49 (24)
Upper secondary		61 (30)
Post-secondary		83 (41)
Missing		12 (6)
**Co-morbidity**		
**Cardiovascular disease, *n* (%)**		
Yes	71 (18)	26 (13)
No	303 (78)	175 (85)
Unknown	14 (4)	4 (2)
**Cerebrovascular disease, *n* (%)**		
Yes	30 (8)	4 (2)
No	349 (90)	199 (97)
Unknown	9 (2)	2 (1)
**Renal dysfunction, *n* (%)**		
Yes	13 (3)	2 (1)
No	363 (94)	200 (98)
Unknown	12 (3)	3 (1)
**Diabetes, *n* (%)**		
Yes	33 (9)	14 (7)
No	346 (89)	190 (93)
Unknown	9 (2)	1
**HIV-positive, *n* (%)**		
Yes	4 (1)	2 (1)
No	373 (96)	201 (98)
Unknown	11 (3)	2 (1)
**Tumour staging, *n* (%)**		
0–II	169 (44)	113 (55)
III–IV	181 (47)	87 (42)
Unknown	38 (10)	5 (2)
**Treatment strategy, *n* (%)**		
Curative	338 (87)	203 (99)
Palliative	44 (11)	0
Unknown	6 (2)	2 (1)
**Recurrence, *n* (%)**		
Yes	76 (20)	28 (14)
No	256 (66)	173 (84)
Unknown	56 (14)	4 (2)

* Variables marital status and educational level were acquired from the questionnaires and can therefore only be presented for the responders. IQR: interquartile range.

High QoL was reported in 40% of responders when measured at 3 and 6 years from diagnosis (76/188 vs. 61/149). About 80% of all responders, irrespective of tumour stage were either moderately or very sure about cure (‘strong perception of cure’) at both 3 and 6 years (Table 2). Notably, only about one third of responders were very sure of cure at both assessments (64/191 vs. 44/154). Perception of cure was relatively stable over time when analysing results for the 144 individuals who had responded to both questionnaires (Table 3).

**Table 2:** Assessment of perception of cure in individuals who responded to the 3- and/or 6-year questionnaire.

Follow-up	Perception of cure				
	Not at all sure	Somewhat sure	Moderately sure	Very sure	Total
3 years, *n* (%)	16 (8)	21 (11)	90 (47)	64 (34)	191 (100)
6 years, *n* (%)	13 (8)	12 (8)	85 (55)	44 (29)	154 (100)

**Table 3:** Assessment of perception of cure in individuals who have responded to both the 3- and 6-year questionnaire.

Follow-up	Perception of cure				
	Not at all sure	Somewhat sure	Moderately sure	Very sure	Total
3 years, *n* (%)	12 (8)	15 (10)	68 (47)	49 (34)	144 (100)
6 years, *n* (%)	10 (7)	9 (6)	82 (57)	43 (30)	144 (100)

Patients with a high QoL had a strong perception of cure (Table 4). A strong perception of cure had a positive impact on QoL in both the unadjusted and adjusted model (Table 5). In the adjusted model SOC and depression were associated with QoL, while other factors were insignificant.

**Table 4:** Patient-reported perception of cure and quality of life among all responders.

Perception of cure	Quality of life					
	3 years			6 years		
	High	Low	Total	High	Low	Total
Not at all sure	2 (13%)	14 (88%)	16 (100%)	1 (8%)	12 (92%)	13 (100%)
Somewhat sure	5 (24%)	16 (76%)	21 (100%)	7 (58%)	5 (42%)	12 (100%)
Moderately sure	32 (37%)	55 (63%)	87 (100%)	30 (37%)	51 (63%)	81 (100%)
Very sure	37 (61%)	24 (39%)	61 (100%)	23 (55%)	19 (45%)	42 (100%)
Total	76 (41%)	109 (59%)	185 (100%)	61 (41%)	87 (59%)	148 (100%)

**Table 5:** Unadjusted and adjusted odds ratios for high QoL.

Perception of cure	OR*	95% CI	*P*
Unadjusted			
Somewhat sure vs. Not at all sure	5.94	(1.11, 31.76)	0.04
Moderately sure vs. Not at all sure	5.80	(1.36, 24.71)	0.02
Very sure vs. Not at all sure	19.10	(4.06, 89.98)	< 0.001
Adjusted			
Somewhat sure vs. Not at all sure	6.89	(1.08, 43.77)	0.04
Moderately sure vs. Not at all sure	2.59	(0.53, 12.62)	0.24
Very sure vs. Not at all sure	6.08	(1.13, 32.62)	0.04

* The odds ratios were based on the mixed-effect logistic regression with quality of life as outcome and the perception of cure as the key exposure variable. The model was further adjusted for bother due to bowel function, sense of coherence, depression and recurrence. QoL: quality of life; CI: confidence interval; OR: odds ratio.

**Figure 2:**
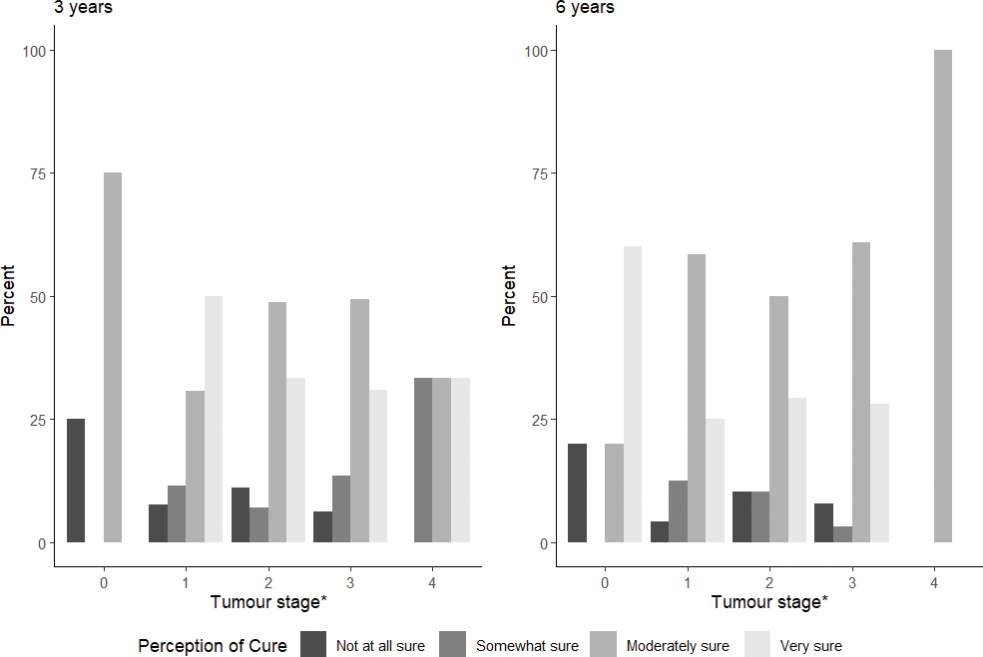
Patient-reported perception of cure and tumour staging. *Tumour stage according to AJCC/UICC seventh edition.

Tumour stage did not seem to be important for the patient’s perception of cure (Figure 2). About 80% seemed to be either moderately or very sure of cure at both 3- and 6- year follow-up in both early and advanced tumour stages (stages 0–2 vs. 3–4).

Even though only 16% had a poor perception of cure (not at all sure or somewhat sure), 63/150 (42%) experienced fear of recurrence 6 years after diagnosis.

Of the remaining patients, 47/150 (31%) were not worried about recurrence while 40/150 (27%) were unsure.

## Discussion and conclusion

In this longitudinal study of a national cohort of patients with anal cancer, we found that a strong perception of cure was associated with a high QoL. Still, even though most survivors from anal cancer reported a perception of being cured, a considerable proportion distrusted the possibility of cure and experienced fear of recurrence several years after treatment.

The prognosis of anal cancer is considered very good. In this national cohort of patients treated at multiple sites with various treatment protocols the 5-year overall survival was 73% for patients treated with curative intent [20]. Since then, national treatment guidelines, have been established and implemented in Sweden with a 3-year overall survival of 85% and most recurrences are found within the first 3 years [3, 4]. All data indicate a high probability of cure after treatment for anal cancer [2, 4, 30–34].

Interestingly, tumour stage did not affect the perception of cure. Our study found that about 80% of survivors, irrespective of tumour stage, expressed a strong perception of cure 3 years after treatment and that it remained stable at the 6-year follow-up. Even though health care professionals know that the risk of recurrence decreases over time, it might be that patients are unaware of this.

Only one third of anal cancer survivors in this cohort were very sure of cure after completed curative treatment and about 40% expressed fear of recurrence after 6 years. This consequently means that anal cancer survivors worry about recurrence after cessation of clinical controls. With our knowledge about anal cancer and its effective treatment, we expected patients to be more confident in being cured at 6 years. Therefore, we find it troublesome that patients seem to have insufficient knowledge about their disease and its good prognosis. We can only speculate that it could be due to a lack of receiving and/or retrieving information.

Other studies of patients with different cancer types and with treatment aiming for cure have shown a perception of cure corresponding to 80–90% [12, 13, 35]. This corresponds well to our data.

Studies have also suggested that the expectation of cure corresponds with clinical tumour staging, indicating that a more advanced cancer disease is associated with more insecurity regarding perceived cure [13, 36]. This contrasts with our study, which could be not only due to our small cohort size but also due to patients lacking knowledge of the association between clinical tumour staging and prognosis.

However, none of the above-mentioned studies have reported perceived oncological outcome and QoL years after finalised treatment. In this perspective, our study stands out.

Prognostic awareness has been examined in patients with incurable cancer. Studies have demonstrated that patients treated with palliative intent often mistake the treatment purpose for being curative and thus have inaccurate expectations about the effectiveness of cancer treatments [6–8, 12, 37–39]. It is not unreasonable to imagine that misconceptions regarding survival and prognosis could be an issue among cancer survivors as well. In fact, we can now report that misjudgements occur among anal cancer survivors and that this leads to overly negative expectations of cure.

Although our study cannot explain why some patients experience insecurity regarding cure, it is possible that if the physician actively gives the patient positive affirmation on being cured, it could improve the perception of cure.

Fear of cancer recurrence was common (42%) at 6 years and occurred not only in patients reporting a poor perception of cure (16%). Our study did not investigate the exact distribution of fear of recurrence among survivors with different perceptions of cure, but we suspect this could be more frequent in patients who feel less sure of being cured.

When evaluating QoL in long-term survivors, one should bear in mind that fear of recurrence appears to be negatively associated with QoL [17, 19]. Reviews indicate that fear of recurrence affects survivors irrespective of cancer type [17, 18]. Surprisingly, the actual occurrence of recurrence did not seem to affect QoL in our population.

Our study has several strengths. Results are derived from a national, unselected cohort including a relatively large number of patients considering the rarity of the disease. This longitudinal study has also successfully achieved a high response rate providing patient-reported outcomes during a long-term follow-up. A major strength is that we have presented information regarding a significant topic that, to the best of our knowledge, has not been explored before.

A limitation of the study was the lack of an established disease-specific questionnaire. There are some tools for measuring prognostic awareness and fear of recurrence, but no established one for either entity [17, 19, 40]. Altogether, this somewhat limits the interpretation of results.

The study cohort was not selected solely to explore these specific objectives, and consequently future assessments can be improved. For instance, the study did not include questions about patient perceived information regarding prognosis from healthcare professionals. Still, our study implies the necessity of developing our knowledge since there is an apparent deficit in the literature.

The study was conducted before the implementation of national treatment guidelines and centralisation of treatment and follow-up in Sweden in 2017. Patients in this cohort have visited many different hospitals and physicians at various time intervals, which could mean that our results do not directly reflect the current situation. Anyhow, with now a limited number of physicians caring for these patients we could have the possibility to influence the information that is given.

In conclusion, our study found that many anal cancer survivors reported a strong perception of cure and that this was related to a high QoL. Still, a substantial number of patients experienced unwarranted insecurity regarding curability. We suggest that it is essential to address the topic of prognosis early and repeatedly during the cancer trajectory.

## Supplementary Material



## Data Availability

The data are available on request after contact with the corresponding author. Due to ethical considerations regarding patient privacy, the data are not publicly shared.

## References

[1] Rao S, Guren MG, Khan K, Brown G, Renehan AG, Steigen SE, et al. Anal cancer: ESMO Clinical Practice Guidelines for diagnosis, treatment and follow-up. Ann Oncol. 2021;32(9):1087–100. 10.1016/j.annonc.2021.06.015 34175386

[2] James RD, Glynne-Jones R, Meadows HM, Cunningham D, Myint AS, Saunders MP, et al. Mitomycin or cisplatin chemoradiation with or without maintenance chemotherapy for treatment of squamous-cell carcinoma of the anus (ACT II): a randomised, phase 3, open-label, 2 × 2 factorial trial. Lancet Oncol. 2013;14(6):516–24. 10.1016/S1470-2045(13)70086-X 23578724

[3] Johnsson A, Norman D, Angenete E, Cavalli-Björkman N, Lagerbäck C, Leon O, et al. Anal cancer in Sweden 2015–2019. Implementation of guidelines, structural changes, national registry and early results. Acta Oncol. 2022;61(5):575–82. 10.1080/0284186X.2022.2048069 35274596

[4] Bentzen AG, Guren MG, Wanderås EH, Frykholm G, Tveit KM, Wilsgaard T, et al. Chemoradiotherapy of anal carcinoma: survival and recurrence in an unselected national cohort. Int J Radiat Oncol Biol Phys. 2012;83(2):e173–80. 10.1016/j.ijrobp.2011.12.062 22436791

[5] Applebaum AJ, Kolva EA, Kulikowski JR, Jacobs JD, DeRosa A, Lichtenthal WG, et al. Conceptualizing prognostic awareness in advanced cancer: a systematic review. J Health Psychol. 2014;19(9):1103–19. 10.1177/1359105313484782 24157936 PMC4665620

[6] Weeks JC, Catalano PJ, Cronin A, Finkelman MD, Mack JW, Keating NL, et al. Patients’ expectations about effects of chemotherapy for advanced cancer. N Engl J Med. 2012;367(17):1616–25. 10.1056/NEJMoa1204410 23094723 PMC3613151

[7] El-Jawahri A, Traeger L, Park ER, Greer JA, Pirl WF, Lennes IT, et al. Associations among prognostic understanding, quality of life, and mood in patients with advanced cancer. Cancer. 2014;120(2):278–85. 10.1002/cncr.28369 24122784

[8] Nipp RD, Greer JA, El-Jawahri A, Moran SM, Traeger L, Jacobs JM, et al. Coping and prognostic awareness in patients with advanced cancer. J Clin Oncol. 2017;35(22):2551–7. 10.1200/JCO.2016.71.3404 28574777 PMC5536163

[9] Zijlstra M, van Roij J, Henselmans I, van Laarhoven HWM, Creemers GJ, Vreugdenhil G, et al. Perception of prognosis and health-related quality of life in patients with advanced cancer: results of a multicentre observational study (eQuiPe). Support Care Cancer. 2023;31(3):165. 10.1007/s00520-023-07631-8 36781515

[10] Bergerot CD, Bergerot PG, Philip EJ, Hsu JA, Dizman N, Vaishampayan U, et al. Perception of cure among patients with metastatic genitourinary cancer initiating immunotherapy. J Immunother Cancer. 2019;7(1):71. 10.1186/s40425-019-0557-5 30867071 PMC6416952

[11] Soylu C, Babacan T, Sever AR, Altundag K. Patients’ understanding of treatment goals and disease course and their relationship with optimism, hope, and quality of life: a preliminary study among advanced breast cancer outpatients before receiving palliative treatment. Support Care Cancer. 2016;24(8):3481–8. 10.1007/s00520-016-3182-6 27003902

[12] Mackillop WJ, Stewart WE, Ginsburg AD, Stewart SS. Cancer patients’ perceptions of their disease and its treatment. Br J Cancer. 1988;58(3):355–8. 10.1038/bjc.1988.218 2460120 PMC2246585

[13] Kim Y, Winner M, Page A, Tisnado DM, Martinez KA, Buettner S, et al. Patient perceptions regarding the likelihood of cure after surgical resection of lung and colorectal cancer. Cancer. 2015;121(20):3564–73. 10.1002/cncr.29530 26094729 PMC4872514

[14] Sodergren SC, Vassiliou V, Dennis K, Tomaszewski KA, Gilbert A, Glynne-Jones R, et al. Systematic review of the quality of life issues associated with anal cancer and its treatment with radiochemotherapy. Support Care Cancer. 2015;23(12):3613–23. 10.1007/s00520-015-2879-2 26289529

[15] Sterner A, Derwinger K, Staff C, Nilsson H, Angenete E. Quality of life in patients treated for anal carcinoma – a systematic literature review. Int J Colorectal Dis. 2019;34(9):1517–28. 10.1007/s00384-019-03342-x 31324957

[16] Axelsson A, Johansson M, Bock D, Haglind E, de la Croix H, Nilsson PJ, et al. Patient-reported QoL in anal cancer survivors 3 and 6 years after treatment-results from the Swedish national ANCA study. Support Care Cancer. 2022;30(5):4169–78. 10.1007/s00520-021-06769-7 35079906 PMC8942973

[17] Simard S, Thewes B, Humphris G, Dixon M, Hayden C, Mireskandari S, et al. Fear of cancer recurrence in adult cancer survivors: a systematic review of quantitative studies. J Cancer Surviv. 2013;7(3):300–22. 10.1007/s11764-013-0272-z 23475398

[18] Luigjes-Huizer YL, Tauber NM, Humphris G, Kasparian NA, Lam WWT, Lebel S, et al. What is the prevalence of fear of cancer recurrence in cancer survivors and patients? A systematic review and individual participant data meta-analysis. Psychooncology. 2022;31(6):879–92. 10.1002/pon.5921 35388525 PMC9321869

[19] Koch L, Jansen L, Brenner H, Arndt V. Fear of recurrence and disease progression in long-term (≥ 5 years) cancer survivors – a systematic review of quantitative studies. Psychooncology. 2013;22(1):1–11. 10.1002/pon.3022 22232030

[20] Johansson M, Axelsson A, Haglind E, Bock D, Angenete E. Long-term survival after treatment for primary anal cancer- results from the Swedish national ANCA cohort study. Acta Oncol. 2022;61(4):478–83. 10.1080/0284186X.2022.2033314 35098862

[21] Steineck G, Helgesen F, Adolfsson J, Dickman PW, Johansson JE, Norlén BJ, et al. Quality of life after radical prostatectomy or watchful waiting. N Engl J Med. 2002;347(11):790–6. 10.1056/NEJMoa021483 12226149

[22] Asplund D, Heath J, González E, Ekelund J, Rosenberg J, Haglind E, et al. Self-reported quality of life and functional outcome in patients with rectal cancer – QoLiRECT. Dan Med J. 2014;61(5):A4841. 24814743

[23] Antonovsky A. The structure and properties of the sense of coherence scale. Soc Sci Med. 1993;36(6):725–33. 10.1016/0277-9536(93)90033-Z 8480217

[24] Axelsson A, Johansson M, Haglind E, Li Y, Nilsson PJ, Angenete E. Patient reported long-term side effects from treatment on urinary and sexual function in anal cancer survivors – 3- and 6-year results from the Swedish national ANCA study. Colorectal Dis. 2024;26(7):1359–69. 10.1111/codi.17040 38816903

[25] Axelsson A, Johansson M, Haglind E, Li Y, Nilsson PJ, Angenete E. Patient reported long-term side effects on bowel function and anal pain in anal cancer survivors – 3- and 6-year results from the Swedish national ANCA study. Colorectal Dis. 2024;26(1):54–62. 10.1111/codi.16814 38010060

[26] Eriksson M, Lindström B. Antonovsky’s sense of coherence scale and its relation with quality of life: a systematic review. J Epidemiol Community Health. 2007;61(11):938–44. 10.1136/jech.2006.056028 17933950 PMC2465600

[27] Asplund D, Bisgaard T, Bock D, Burcharth J, González E, Haglind E, et al. Pretreatment quality of life in patients with rectal cancer is associated with intrusive thoughts and sense of coherence. Int J Colorectal Dis. 2017;32(11):1639–47. 10.1007/s00384-017-2900-y 28913686 PMC5635091

[28] Vähäaho N, Hakamies-Blomqvist L, Blomqvist C, Kellokumpu-Lehtinen PL, Huovinen R, Saarto T, et al. Sense of coherence as predictor of quality of life in early breast cancer patients. Anticancer Res. 2021;41(10):5045–52. 10.21873/anticanres.15319 34593453

[29] Skoogh J, Ylitalo N, Larsson Omeróv P, Hauksdóttir A, Nyberg U, Wilderäng U, et al. ‘A no means no’ – measuring depression using a single-item question versus Hospital Anxiety and Depression Scale (HADS-D). Ann Oncol. 2010;21(9):1905–9. 10.1093/annonc/mdq058 20231301

[30] Ajani JA, Winter KA, Gunderson LL, Pedersen J, Benson AB, 3rd, Thomas CR, Jr., et al. Fluorouracil, mitomycin, and radiotherapy vs fluorouracil, cisplatin, and radiotherapy for carcinoma of the anal canal: a randomized controlled trial. JAMA. 2008;299(16):1914–21. 10.1001/jama.299.16.1914 18430910

[31] Northover J, Glynne-Jones R, Sebag-Montefiore D, James R, Meadows H, Wan S, et al. Chemoradiation for the treatment of epidermoid anal cancer: 13-year follow-up of the first randomised UKCCCR Anal Cancer Trial (ACT I). Br J Cancer. 2010;102(7):1123–8. 10.1038/sj.bjc.6605605 20354531 PMC2853094

[32] Slørdahl KS, Klotz D, Olsen J, Skovlund E, Undseth C, Abildgaard HL, et al. Treatment outcomes and prognostic factors after chemoradiotherapy for anal cancer. Acta Oncol. 2021;60(7):921–30. 10.1080/0284186X.2021.1918763 33966592

[33] Leon O, Guren M, Hagberg O, Glimelius B, Dahl O, Havsteen H, et al. Anal carcinoma – survival and recurrence in a large cohort of patients treated according to Nordic guidelines. Radiother Oncol. 2014;113(3):352–8. 10.1016/j.radonc.2014.10.002 25499203

[34] Glynne-Jones R, Sebag-Montefiore D, Meadows HM, Cunningham D, Begum R, Adab F, et al. Best time to assess complete clinical response after chemoradiotherapy in squamous cell carcinoma of the anus (ACT II): a post-hoc analysis of randomised controlled phase 3 trial. Lancet Oncol. 2017;18(3):347–56. 10.1016/S1470-2045(17)30071-2 28209296 PMC5337624

[35] Winner M, Wilson A, Yahanda A, Kim Y, Pawlik TM. A cross-sectional study of patient and provider perception of ‘cure’ as a goal of cancer surgery. J Surg Oncol. 2016;114(6):677–83. 10.1002/jso.24401 27696412

[36] Agarwal R, Shin P, Knezevic A, Nelson JE, Romano DR, Bernal C, et al. Accuracy of curability expectations in patients with gastrointestinal cancers. Cancer Med. 2023;12(1):20–9. 10.1002/cam4.4947 35959986 PMC9844646

[37] Craft PS, Burns CM, Smith WT, Broom DH. Knowledge of treatment intent among patients with advanced cancer: a longitudinal study. Eur J Cancer Care (Engl). 2005;14(5):417–25. 10.1111/j.1365-2354.2005.00601.x 16274462

[38] Yennurajalingam S, Rodrigues LF, Shamieh O, Tricou C, Filbet M, Naing K, et al. Perception of curability among advanced cancer patients: an international collaborative study. Oncologist. 2018;23(4):501–6. 10.1634/theoncologist.2017-0264 29158371 PMC5896700

[39] Minichsdorfer C, Zeller O, Kirschbaum M, Berghoff AS, Bartsch R. Expectations and perception of cancer treatment goals in previously untreated patients. The EXPECT trial. Support Care Cancer. 2021;29(7):3585–92. 10.1007/s00520-020-05826-x 33159221 PMC8163685

[40] Luciani F, Veneziani G, Giraldi E, Campedelli V, Galli F, Lai C. To be aware or not to be aware of the prognosis in the terminal stage of cancer? A systematic review of the associations between prognostic awareness with anxiety, depression, and quality of life according to cancer stage. Clin Psychol Rev. 2025;116:102544. 10.1016/j.cpr.2025.102544 39809049

