# Supplementary material for: Perception of cure and quality of life in anal cancer survivors
Source: Acta Oncol. 2025 Oct 15;64:44133. doi: 10.2340/1651-226X.2025.44133 (PMC12534994; doi:10.2340/1651-226X.2025.44133)
Supplement: Supplementary file 1 [file AO-64-44133-s1.pdf]

Supplementary material has been published as submitted. It has not been copyedited, or typeset by Acta Oncologica

Table 6. Patient and tumour characteristics of responders vs. non-responders

Characteristic	Responders 3 and/or 6 years	Deceased or non-responders
**Age, median (IQR)**	64 (58, 71)	70 (59, 82)
**Sex, n (%)**		
Male	46 (22)	61 (33)
Female	159 (78)	122 (67)
**Cardiovascular disease, n (%)**		
Yes	26 (13)	45 (25)
No	175 (85)	128 (70)
Unknown	4 (2)	10 (5)
**Cerebrovascular disease, n (%)**		
Yes	4 (2)	26 (14)
No	199 (97)	150 (82)
Unknown	2 (1)	7 (4)
**Renal dysfunction, n (%)**		
Yes	2 (1)	11 (6)
No	200 (98)	163 (89)
Unknown	3 (1)	9 (5)
**Diabetes, n (%)**		
Yes	14 (7)	19 (10)
No	190 (93)	156 (85)
Unknown	1	8 (4)
**HIV-positive, n (%)**		
Yes	2 (1)	2 (1)
No	201 (98)	172 (94)
Unknown	2 (1)	9 (5)
**Tumour staging, n (%)**		
0-II	113 (55)	56 (31)
III-IV	87 (42)	94 (51)
Unknown	5 (2)	33 (18)
**Treatment strategy, n (%)**		
Curative	203 (99)	135 (74)
Palliative	0	44 (24)
Unknown	2 (1)	4 (2)
**Recurrence, n (%)**		
Yes	28 (14)	48 (26)
No	173 (84)	83 (45)
Unknown	4 (2)	52 (29)
**Type of treatment, n (%)**		
Chemotherapy followed by radiotherapy	40 (20)	26 (14)
Chemoradiotherapy	106 (52)	49 (27)

Radiotherapy	46 (22)	74 (40)
Chemotherapy	0	6 (3)
Surgery	9 (4)	7 (4)
Surgery followed by adjuvant	3 (2)	5 (3)
Chemotherapy followed by surgery	1	0
Best supportive care	0	15 (8)
Unknown	0	1
